# Supplementary material for: Stochastic Gene Expression Influences the Selection of Antibiotic Resistance Mutations
Source: Mol Biol Evol. 2019 Sep 4;37(1):58–70. doi: 10.1093/molbev/msz199 (PMC6984361; doi:10.1093/molbev/msz199)
Supplement: msz199_Supplementary_Data [file msz199_supplementary_data.zip › msz199-Suppl_data/supplementary_material.pdf]

Supplementary Material

Stochastic gene expression influences the selection of antibiotic resistance mutations

Lei Sun, Peter Ashcroft, Martin Ackermann, and Sebastian Bonhoeffer

S.I Mean field model

From the reactions in Table 2 of the manuscript, we can construct a mean-field ordinary differential equation (ODE) model:

$$\dot{P}_{\mathrm{T}} = \Gamma - k_f \frac{P_{\mathrm{T}}\,\phi}{V(t)} + k_b \theta_{\mathrm{T}}, \tag{S1a}$$

$$\dot{D}_{\mathrm{E}} = \Gamma_{\mathrm{On}}(1 - D_{\mathrm{E}}) - \Gamma_{\mathrm{Off}} D_{\mathrm{E}}, \tag{S1b}$$

$$\dot{M}_{\mathrm{E}} = \tau D_{\mathrm{E}} - \gamma M_{\mathrm{E}}, \tag{S1c}$$

$$\dot{P}_{\mathrm{E}} = \gamma b M_{\mathrm{E}} - k_f \frac{P_{\mathrm{E}}\,\phi}{V(t)} + (k_b + k_{\mathrm{cat}})\theta_{\mathrm{E}}, \tag{S1d}$$

$$\dot{\phi} = \sigma A(t) \left[c_{\mathrm{out}} - \frac{\phi}{V(t)} \right] - k_f \frac{(P_{\mathrm{T}} + P_{\mathrm{E}})\phi}{V(t)} + k_b(\theta_{\mathrm{T}} + \theta_{\mathrm{E}}), \tag{S1e}$$

$$\dot{\theta}_{\mathrm{T}} = k_f \frac{P_{\mathrm{T}}\,\phi}{V(t)} - k_b \theta_{\mathrm{T}}, \tag{S1f}$$

$$\dot{\theta}_{\mathrm{E}} = k_f \frac{P_{\mathrm{E}}\,\phi}{V(t)} - (k_b + k_{\mathrm{cat}})\theta_{\mathrm{E}}. \tag{S1g}$$

For the behaviour shown in Fig. 2 of the manuscript, these equations are integrated until time t_G, after which the molecule numbers are halved to represent dilution due to cell division.

S.II Comparing MIC measurements

The population of cells roughly follows the growth law $n(t) = n(0)10^{\psi t}$, where $n(0)$ is the initial population size and ψ is the log-10 growth rate. To find a population growth rate, we then solve this equation for ψ, i.e.,

$$\psi = \frac{\log_{10}[n(t)] - \log_{10}[n(0)]}{t}. \tag{S2}$$

If we measure a lineage extinction probability of X (say, 90%), then the maximum population size at time t that we could observe is $n(t) = (1 - X)n(0)10^{\psi_{\max} t}$, where each of the surviving lineages $(1 - X)$ grow at the maximum rate. Likewise, the minimum population size with a lineage extinction probability of X is $n(t) = (1 - X)n(0)$, i.e. each surviving lineage only has a single cell remaining. We therefore have the following bounds for the growth rate:

$$\frac{\log_{10}(1 - X)}{t} < \psi < \frac{\log_{10}(1 - X)}{t} + \psi_{\max}. \tag{S3}$$

These bounds are highlighted in Supplementary Fig. S1A, from which we see that IC90 measurements after one hour would overestimate the zMIC – instead the IC90 would correspond to a negative net growth rate of the population. When measuring growth rates at later timepoints, we can have correspondence between the IC90 and zMIC, while IC50 is likely to underestimate the concentration which gives a net growth rate of zero.

To confirm the above findings, we compare the ICX values extracted from lineage survival simulations with inhibitory concentrations as measured in growth rate simulations. From the overnight lineage survival probability simulations, we interpolate IC50 (50% lineage survival), IC90 (10% lineage survival), and IC99 (1% lineage survival). From the five hour growth rate

simulations, we interpolate the drug concentrations at which $\psi = 0.1\psi_{\max}$ (10% max growth rate), $\psi = 0.01\psi_{\max}$ (1% max growth rate), and $\psi = 0$ (zero growth, or zMIC). We measure the growth rates at multiple timepoints to determine the observations are dependent on experiment duration. In Supplementary Fig. S1B, we see that the inhibitory concentrations inferred from growth rates converge to IC90 for the REG-ON, REG-OFF, STRUCT-CAT, WT and KO cell types, and this convergence occurs within 3 hours. IC50, on the other hand, always underestimates the true inhibitory concentration. For the STRUCT-BIND and REG-BURST mutants, the inhibitory concentration interpolated from growth rates continually increases with time and IC90 underestimates the zMIC. Therefore IC90 is a conservative estimate of the MIC of the STRUCT-BIND and REG-BURST mutants. In summary, we find that IC90 is the most suitable measure of MIC which generally correlates well with the zMIC drug concentration.

S.III Systematic simulations

Results in the manuscript are based on a single resistance mechanism, the AcrAB-TolC efflux pump, providing resistance to a specific drug, ciprofloxacin. To shed more light on our results, we conduct a systematic sweep of the parameter space to assess in which regimes different mutant classes are advantageous, and what are the maximum levels of resistance that can be observed.

We consider all permutations of the parameters listed in the table below:

Table S1: Summary of parameter values. [a] Average values per generation. [b] Number of efflux pumps is increased by increasing the mRNA burst size b (DNA activation/deactivation and mRNA degradation rates kept constant). [c] Evaluated at 1×MIC.

Parameter	Values
Binding rate ($M^{-1}s^{-1}$)	$10^4, 10^5, 10^6$
Catalysis rate (s^{-1})	$10^{-4}, 10^{-2}, 10$
Drug diffusion rate (ms^{-1})	$10^{-11}, 10^{-10}, 10^{-9}$
Number of targets[a]	$10, 10^2, 10^3$
Number of efflux pumps[a,b]	$10, 10^2, 10^3$
Number of drug molecules[a,c]	$10, 10^2, 10^3$

The three values for each of the six parameters gives a total of $3^6 = 729$ unique parameter combinations. For each of these we consider the WT cell, as well as the REG-ON, STRUCT-BIND, and STRUCT-CAT mutants with an effect of $\mu = 200$.

For each parameter combination, we need to determine the external drug concentration for which the average number of drug molecules per WT cell corresponds to the value in the table above. This concentration is then the MIC of that specific parameter combination. We then use the mean-field model [Eq. (S1)] to calculate the average fraction of bound targets per cell, and we assign this value as the MIC fraction, ρ_{MIC}. We set $\kappa = 3$ throughout, and the remaining parameters are the same as in Supplementary Table S2.

Concretely, for each parameter combination we do the following:

1. Load the basic model parameters, and modify the binding, catalysis, and diffusion rates, and the number of targets directly;

2. Set the rate of efflux mRNA transcription such that the average number per cell matches the designated value;

3. Using these parameters, we integrate the mean-field equations for different external drug concentrations, and solve for an external concentration which gives the average number of drug molecules per cell as given in the table above;

4. We integrate the mean-field equations at this given concentration, and determine the MIC fraction as the average fraction of bound targets.

With this procedure, each of the 729 combinations can be rapidly parametrised, although not completely accurately. We then compute the IC90 values for the each of the $729 \times \{$WT ,REG-ON, STRUCT-BIND, STRUCT-CAT$\}$ combinations, and extract the relative increase in IC90 of each of the mutant cells. The distribution of these mutational effects are shown in Fig. 8 of the manuscript.

To understand when each mutant is advantageous, we can compute the correlation between each of the six parameters and the fold-increase in IC90. This is shown in Fig. S10. A general trend across the three mutants is that resistance is highest when the baseline number of efflux proteins is high, binding rates are fast and diffusion rates are slow.

S.IV Supplementary tables

Model parameters are given in Table S2, and drug-specific parameters are given in Table S3. IC50 and IC90 values for ciprofloxacin treatments can be found in Table S4.

Table S2: Summary of parameter values. [a]Weighted average of reported values. [b]$t_G = \log(2)/(\log(10)\psi_{\max})$. [c]Inactive time reported as 0.5–3,000 min. [d]Active time reported as 5–60 min. [e]Examples range from 5–40.

Parameter	Value	Description
d	934 nm (Ouzounov et al., 2016)	Diameter of cell (constant)
ℓ_0	3.4 μm (Campos et al., 2014)	Length of newborn cell
$\psi_{\max}$	0.80 hour^{-1} (Regoes et al., 2004)[a]	Maximum population growth rate
$\psi_{\min}$	See Table S3	Minimum population growth rate
t_G	22.5 min (Regoes et al., 2004)[b]	Generation time
Γ	See Eq. (2) of the manuscript	Rate of target production
Γ_{On}	0.2 min^{-1} (Lionnet and Singer, 2012; Hammar et al., 2014)[c]	Rate of efflux gene activation
Γ_{Off}	0.005 min^{-1} (Lionnet and Singer, 2012; Hammar et al., 2014)[d]	Rate of efflux gene inactivation
τ	0.42 min^{-1} (Golding et al., 2005)	Transcription rate
γ	0.14 min^{-1} (Bernstein et al., 2002)	mRNA decay rate
b	22.5 (Thattai and Van Oudenaarden, 2001)[e]	Burst size (products per mRNA)
c_{out}	See Table S3	Outside drug concentration
σ	See Table S3	Drug diffusion rate
k_f	See Table S3	Binding rate of drug to target/efflux
k_b	See Table S3	Dissociation rate of drug–protein bound state
k_{cat}	See Table S3	Catalysis/removal rate of drug
MIC	See Table S3	Minimum inhibitory concentration
ρ_{MIC}	See Table S3	MIC fraction of bound targets
κ	See Table S3	Shape parameter
μ	> 1	Mutant effect
ν	5%	Cost of mutation

Table S3: Drug-specific parameters extracted from the literature. [a]Value is based on AcrB efflux pump. [b]Values chosen according to Fig. S2. [c]Values chosen according to Fig. S3.

Drug	Ciprofloxacin	Rifampicin
Target	DNA Gyrase	RNA polymerase
Number of targets	300 (Chong et al., 2014; Stracy et al., 2019)	11,400 (Abel Zur Wiesch et al., 2015)
MIC ($\mu\text{g}\,\text{ml}^{-1}$)	0.03 (Regoes et al., 2004)	8.00 (Regoes et al., 2004)
Molar mass ($\text{g}\,\text{mol}^{-1}$)	331.347	822.953
Diffusion rate ($\text{m}\,\text{s}^{-1}$)	2.0×10^{-11} (Abel Zur Wiesch et al., 2015)	2.0×10^{-11} (Abel Zur Wiesch et al., 2015)
Binding rate ($\text{M}^{-1}\,\text{s}^{-1}$)	3.6×10^{4} (Kampranis and Maxwell, 1998)	1.2×10^{6} (Abel Zur Wiesch et al., 2015)
Dissociation rate (s^{-1})	3.0×10^{-4} (Kampranis and Maxwell, 1998)	1.2×10^{-3} (Abel Zur Wiesch et al., 2015)
Catalysis rate (s^{-1})	10.0 (Nagano and Nikaido, 2009)[a]	10.0 (Nagano and Nikaido, 2009)[a]
Min. pop. growth rate (h^{-1})	-6.5 (Regoes et al., 2004)	-4.3 (Regoes et al., 2004)
Shape (κ)	2.0[b]	2.0[c]
ρ_{MIC}	0.081[b]	0.294[c]

Table S4: Profiles of resistance to ciprofloxacin. The IC50 and IC90 values are expressed as multiples of the MIC for the WT strain. These values are extracted from Fig. 3 of the manuscript using spline interpolation, and are defined as the drug concentrations at which 50% and 90% of the lineages from the initial bacterial inoculum are killed.

Mutant class	Mutant effect (μ)	IC50	IC90
WT	1	0.9	1.0
KO	1	0.9	1.1
REG-ON	2	0.8	1.0
	10	1.0	1.2
	50	1.3	1.5
	200	1.5	1.8
REG-OFF	2	0.8	1.0
	10	0.9	1.2
	50	1.1	1.7
	200	1.3	1.9
REG-BURST	2	0.8	1.0
	10	0.9	1.2
	50	1.1	2.0
	200	1.2	4.5
STRUCT-BIND	2	0.8	1.0
	10	0.9	1.2
	50	1.0	2.0
	200	1.2	4.6
STRUCT-CAT	2–200	0.8	0.9

S.V Supplementary figures

Comparison of MIC measurements is shown in Supplementary Fig. S1. Parameter screening for ciprofloxacin is shown in Supplementary Fig. S2. Parameter screening for rifampicin is shown in Supplementary Fig. S3. Molecule distributions are shown in Supplementary Fig. S4. Results for REG-BURST mutants are shown in Supplementary Fig. S5. Extinction times are shown in Supplementary Fig. S6. Time-kill curves are shown in Supplementary Fig. S7. Results of biasing the binomial distribution of efflux pumps is shown in Supplementary Fig. S8. Survival probabilities following pulsed drug treatment are shown in Supplementary Fig. S9. The correlation between parameter values and mutational effects from the systematic grid sampling is shown in Supplementary Fig. S10.

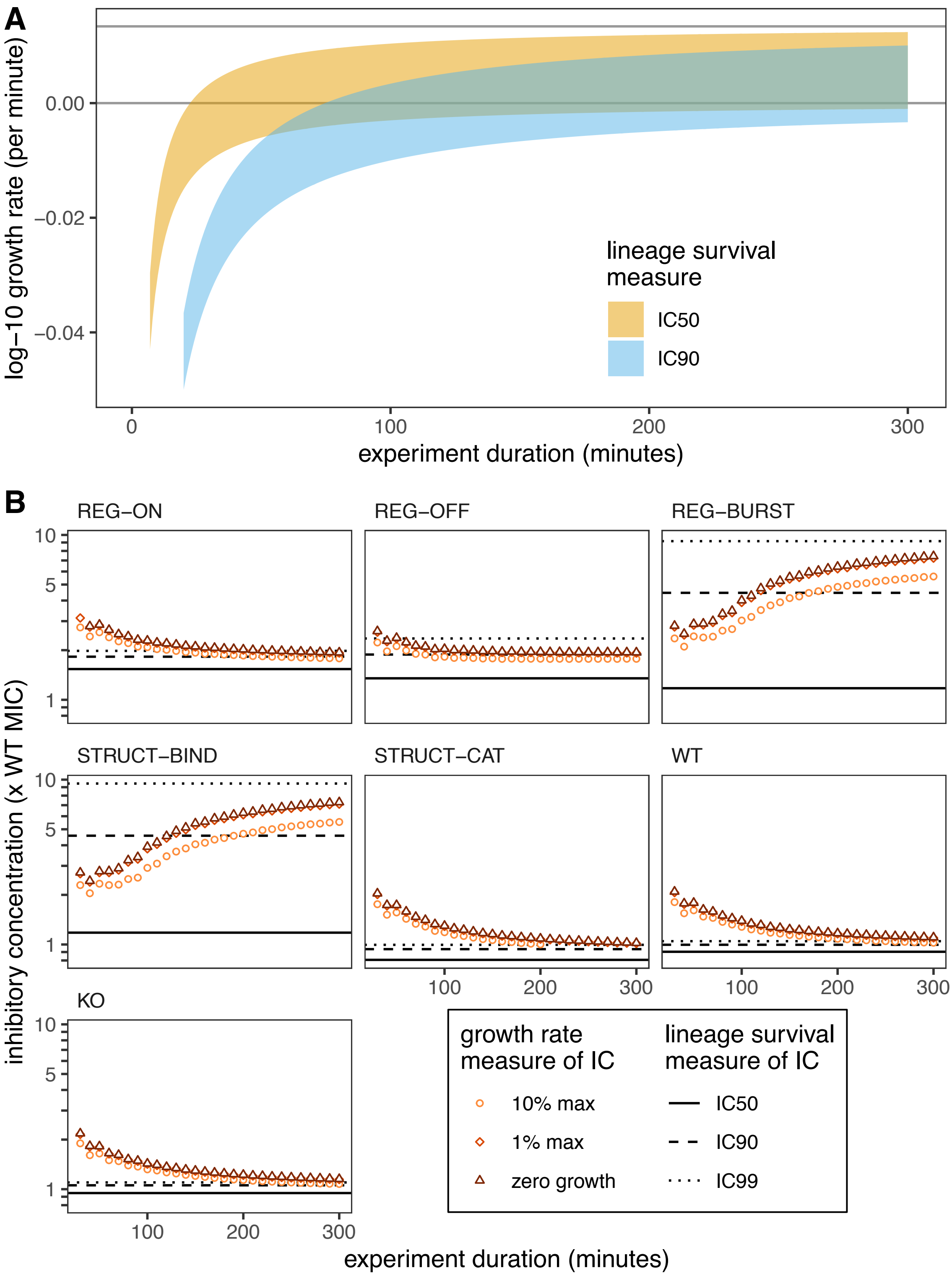

Figure S1: Comparing MIC measurements from growth rate data and lineage survival probabilities. A) Range of growth rates that can be measured for a given lineage survival probability. IC50 has a survival probability of 50%, while IC90 has a survival probability of 10%. Curves are predicted from Eq. (S3). Grey horizontal lines are $\psi_{\max}$ (upper) and zero growth rate. B) Comparison of inhibitory concentrations as determined by lineage survival (lines) or growth rates (symbols). Here we use the parameters for ciprofloxacin. Lineage survival is measured after 1,200 minutes, while growth rates are computed as described in Fig. 4 of the manuscript.

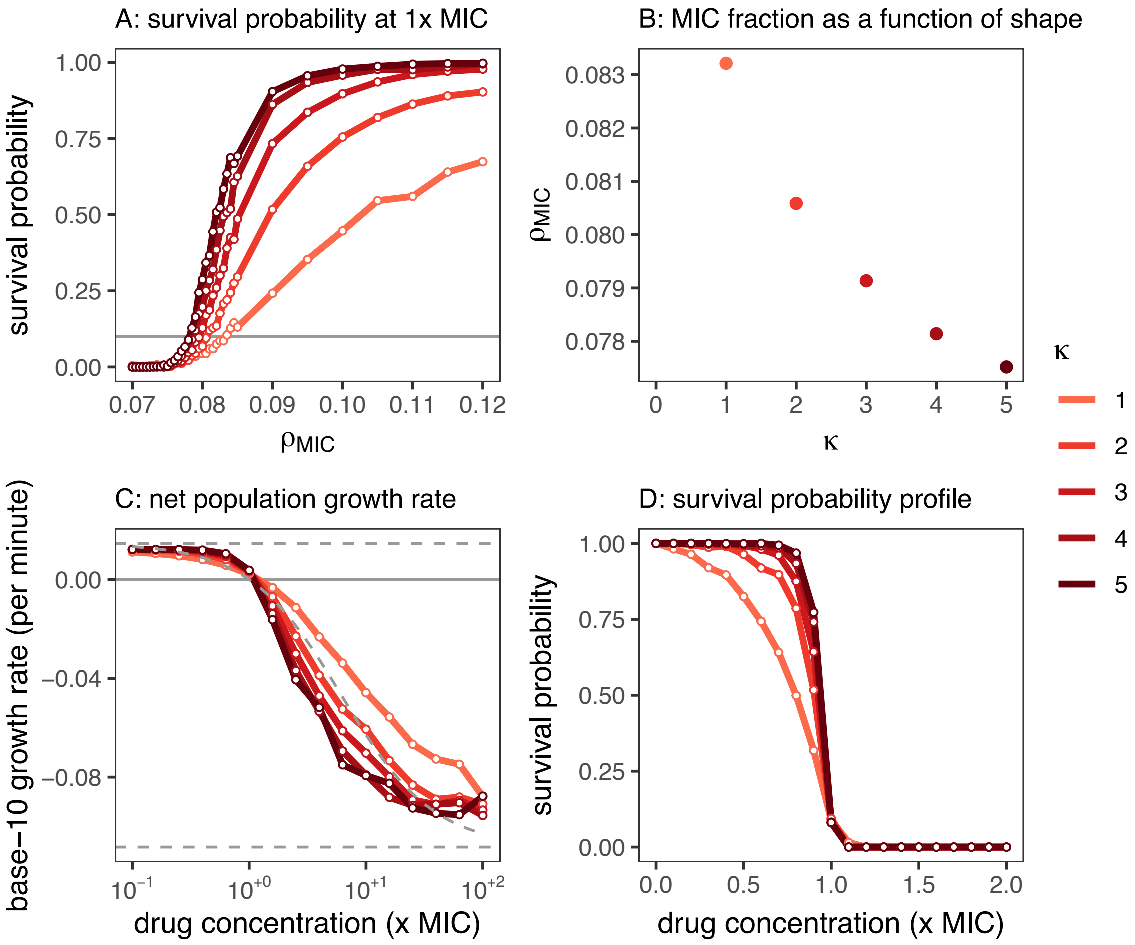

Figure S2: Parameter screening for ciprofloxacin. A) We calculate the survival probability for 1,000 lineages initiated from a single cell at 1×MIC while varying the MIC fraction of bound targets (ρ_{MIC}) and the shape parameter κ. B) From panel A we interpolate the value of ρ_{MIC} which gives a survival probability of 10% (IC90). C) For each pair of κ and ρ_{MIC} in panel B, we simulate a population of 100,000 cells under different drug pressure for three hours, and extract the net population growth rate. This can be compared with the maximum and minimum reported rates (dashed horizontal lines), and the growth curve reported by Regoes et al. (2004) (dashed curve). D) For each pair of κ and ρ_{MIC} in panel B, we calculate the survival probability of 1,000 lineages initiated from a single cell under different drug pressure. From this figure we determine the shape parameter for the death rate of ciprofloxacin is $\kappa = 2$ and the MIC fraction is $\rho_{\text{MIC}} = 0.081$.

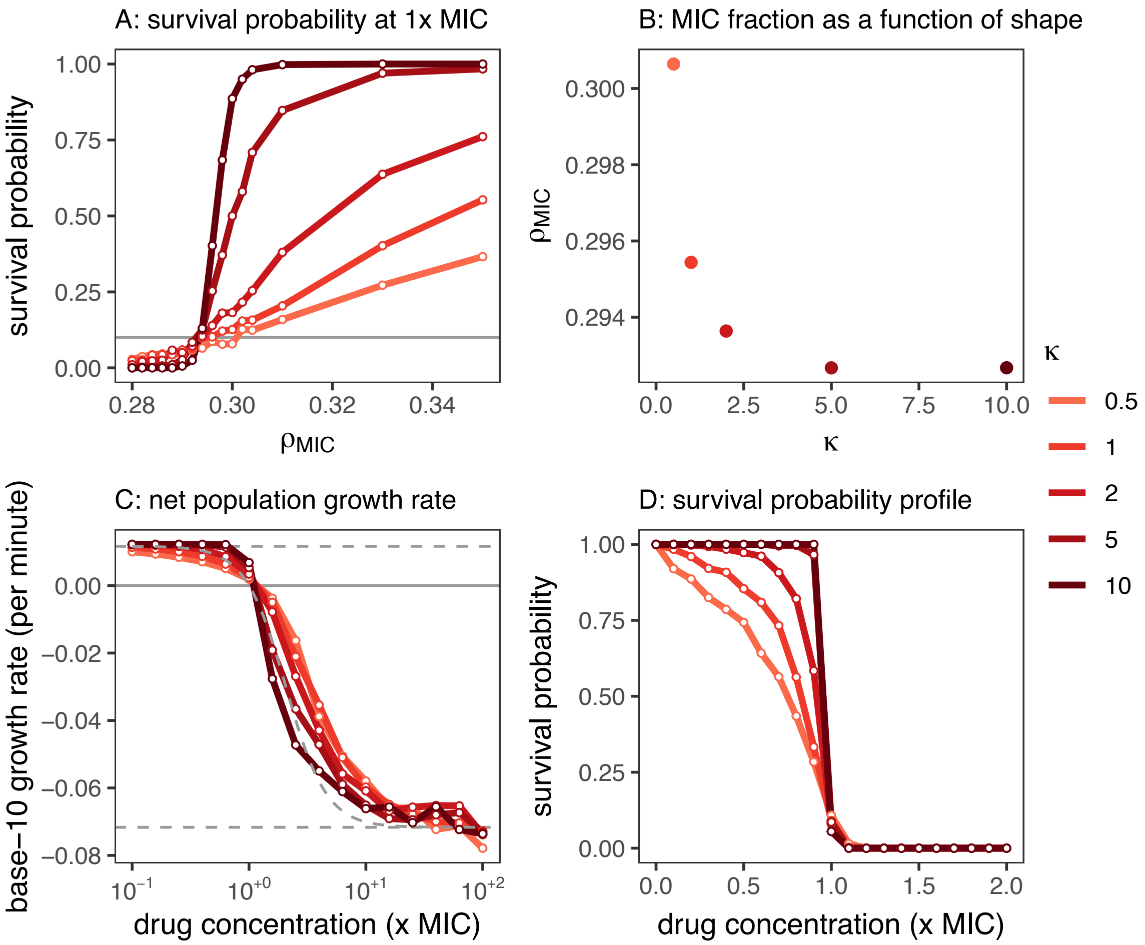

Figure S3: Parameter screening for rifampicin. A) We calculate the survival probability for 1,000 lineages initiated from a single cell at 1×MIC while varying the MIC fraction of bound targets (ρ_{MIC}) and the shape parameter κ. B) From panel A we interpolate the value of ρ_{MIC} which gives a survival probability of 10% (IC90). C) For each pair of κ and ρ_{MIC} in panel B, we simulate a population of 100,000 cells under different drug pressure for three hours, and extract the net population growth rate. This can be compared with the maximum and minimum reported rates (dashed horizontal lines), and the growth curve reported by Regoes et al. (2004) (dashed curve). D) For each pair of κ and ρ_{MIC} in panel B, we calculate the survival probability of 1,000 lineages initiated from a single cell under different drug pressure. From this figure we determine the shape parameter for the death rate of rifampicin is $\kappa = 2$ and the MIC fraction is $\rho_{\text{MIC}} = 0.294$.

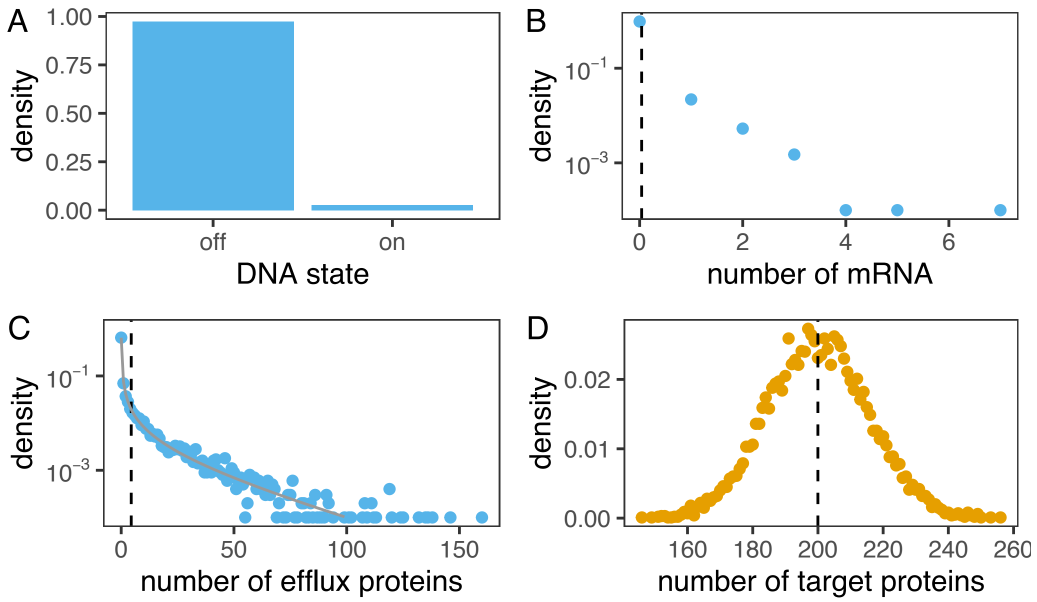

Figure S4: The distribution of efflux and target states across 10,000 cells immediately after cell division. These are sampled after $t = 1,200$ minutes in the absence of drugs. The panels show: A) expression state of the efflux gene; B) distribution of efflux mRNA across the population of cells; C) number of efflux proteins per cell, which fits to a negative binomial distribution (grey line); D) number of target proteins (gyrase) per cell. Parameters are based on the targets of ciprofloxacin, and can be found in Tables S2 and S3.

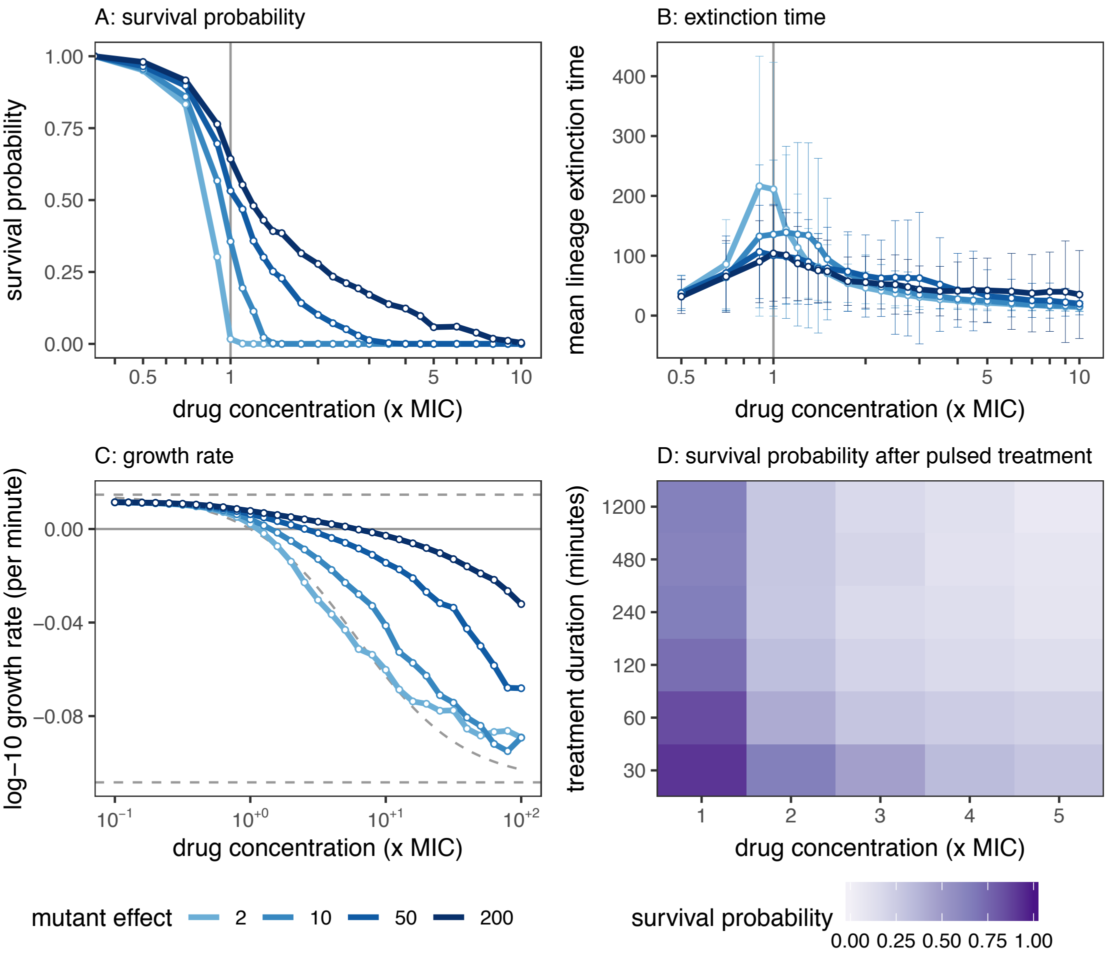

Figure S5: The dynamics of the REG-BURST mutants are very similar to those of STRUCT-BIND. Survival probability (A) and extinction times (B) of the REG-BURST mutants across drug concentrations are calculated as in Fig. 3 of the manuscript and Fig. S6. Colour scale indicates the mutant effect (μ). C) The net population growth rates as computed in Fig. 4 of the manuscript. D) Survival probability after exposure to a pulse of antibiotics for the REG-BURST mutant with 200-fold effect. The survival probability is indicated by colour scale. Simulations are performed as in Fig. 7 of the manuscript and Fig. S9.

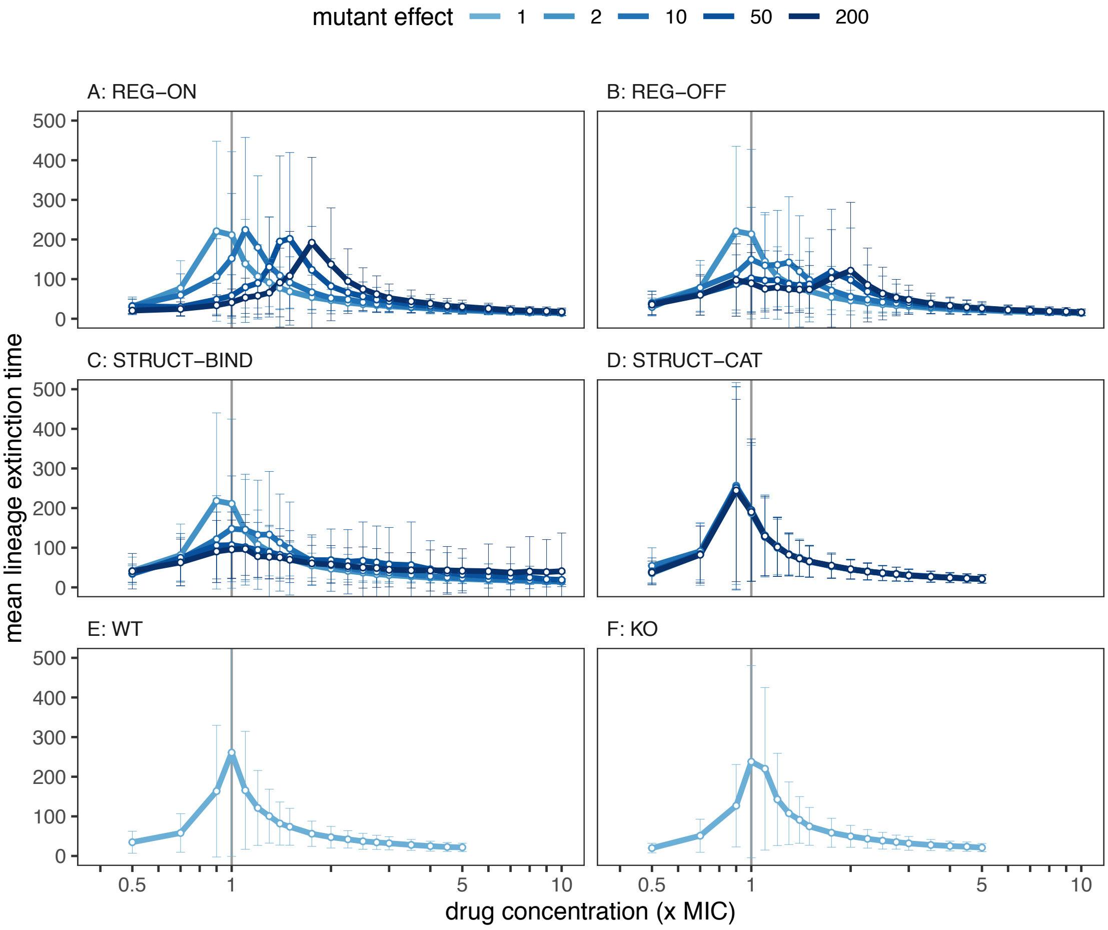

Figure S6: Mean extinction times of lineages of the different mutant classes. These are extracted from the same data as Fig. 3 of the manuscript. Error bars indicate the standard deviation of the extinction times.

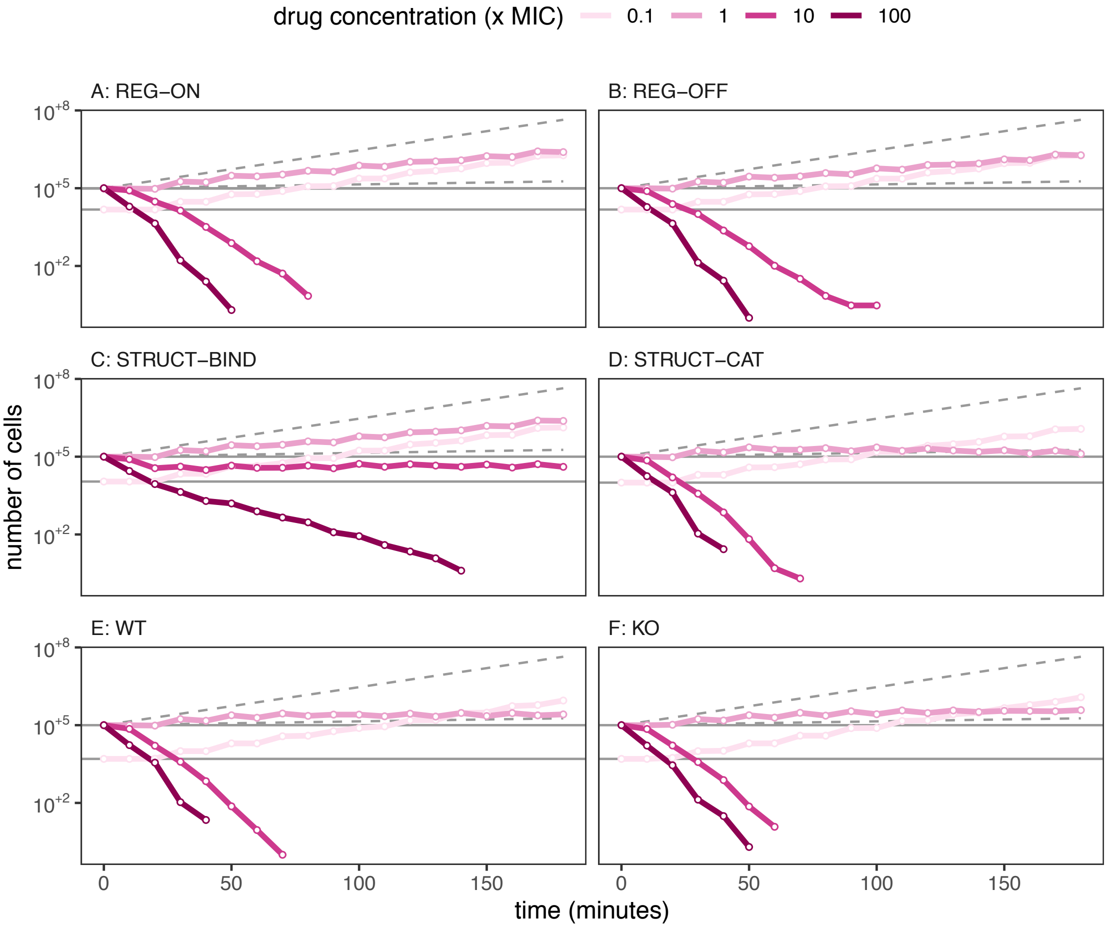

Figure S7: Time-kill curves, from which the growth rates in Fig. 4 of the manuscript are extracted. Here we only show mutant effect parameters $\mu = 200$ (for REG-ON, REG-OFF, STRUCT-BIND, and STRUCT-CAT), and a subset of ciprofloxacin concentrations. Upper dashed line is the projected growth trajectory in the absence of drug, based on the maximum growth rate reported by Regoes et al. (2004). Lower dashed line is the trajectory if the growth rate is reduced by 90% from its maximum value. Horizontal line represents the zero growth scenario. Note that at very low drug concentrations, we reduced the initial size of the population from 10^5 to $\sim 10^4$ due to the computational cost. This has no effect on the measurement of growth rates as the population size never approaches small numbers at these drug concentrations.

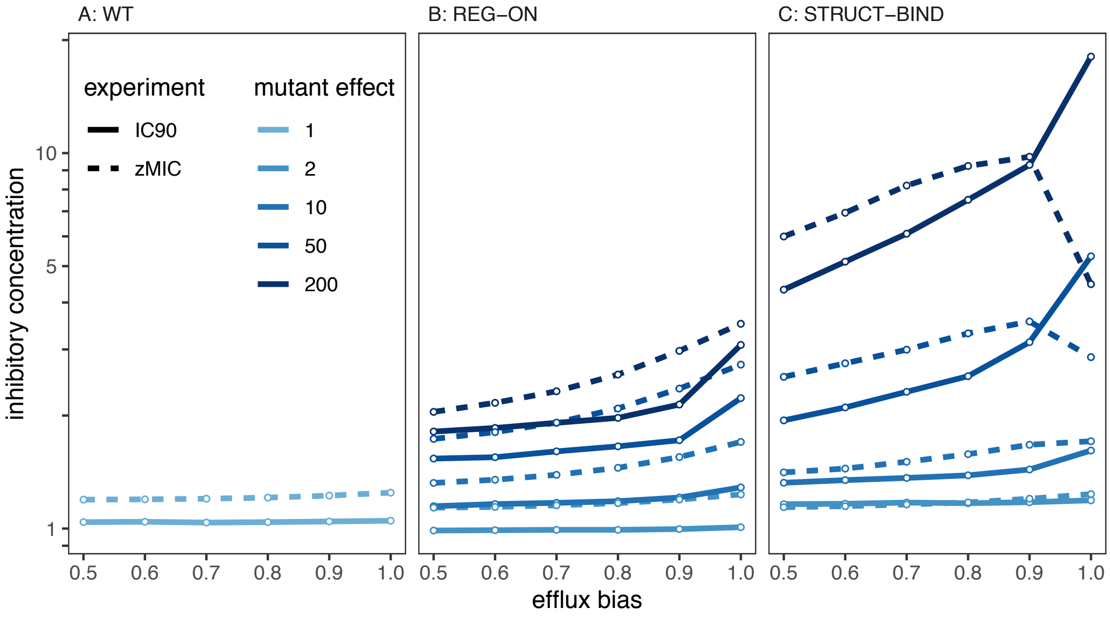

Figure S8: Impact of varying the efflux bias distribution during cell division. The efflux proteins (both bound and unbound) are distributed among the two daughter cells according to a binomial distribution with parameter p, while remaining molecules are distributed with parameter $p = 0.5$. To extract IC90 values, we compute the lineage survival probability of 1,000 initial cells over a range of drug concentrations ($c_{\text{out}} \in [0, 10] \times \text{MIC}$), mutant effects ($\mu \in [1, 200]$), and efflux bias parameters ($p \in [0.5, 1.0]$), for $t = 1,200$ minutes. For zMIC values, we simulated up to 100,000 initial cells and tracked the population size for $t = 180$ minutes over the similar parameter ranges ($c_{\text{out}} \in [10^{-1}, 10^2]$) before extracting the growth rates as in Fig. 4 of the manuscript.

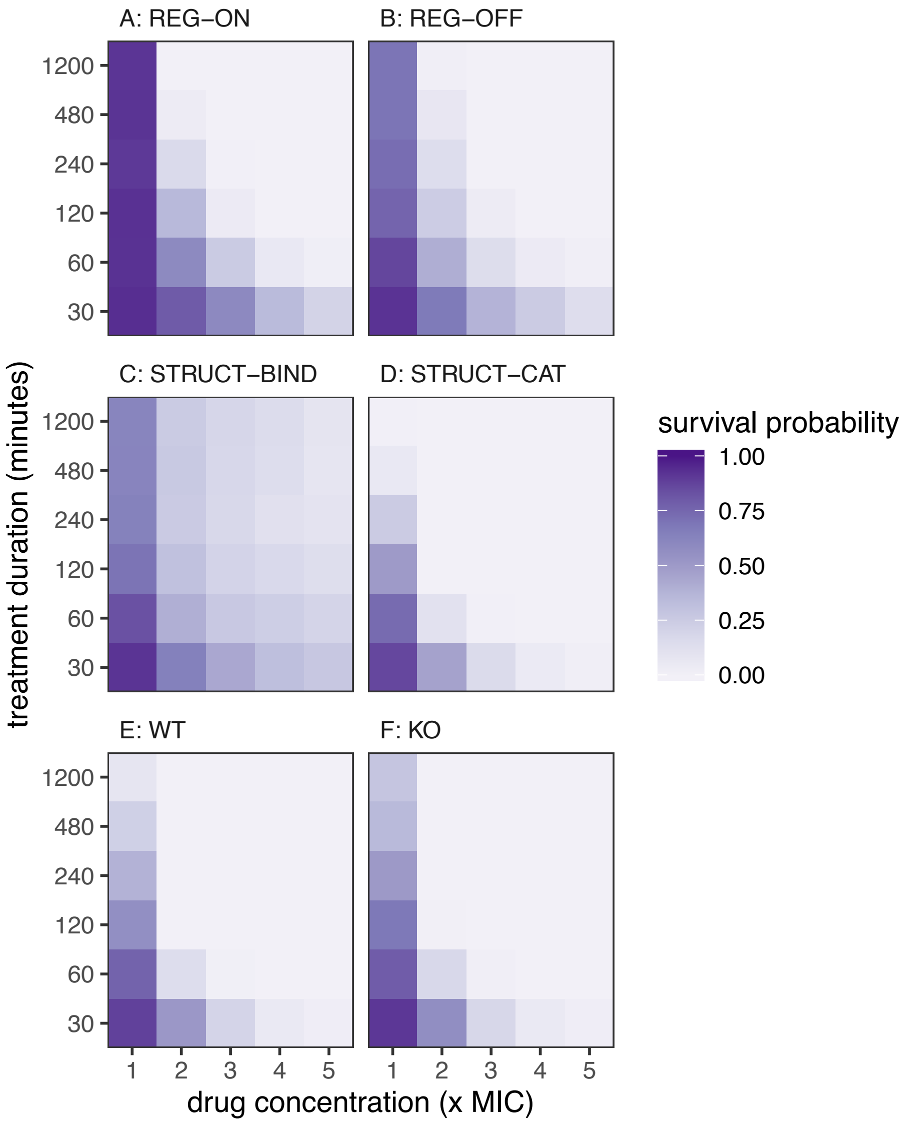

Figure S9: Survival probability after exposure to a pulse of antibiotics. The survival probability is indicated by colour scale. The drug dose is applied as a step-function with constant external drug concentration during the pulse, and $c_{\text{out}} = 0$ for the remainder of the experiment up to $t = 1,200$ minutes. Simulations are performed as in Fig. 3 of the manuscript. The mutant effect is $\mu = 200$.

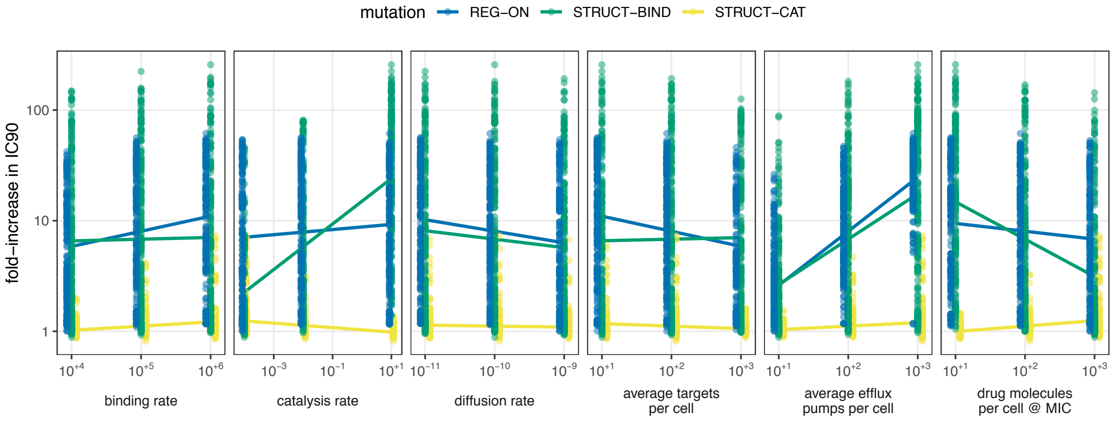

Figure S10: Correlation between parameter values and mutant efficacy following systematic parameter sampling. IC90 is measured for each of the 729 parameter combinations in WT, REG-ON, STRUCT-BIND and STRUCT-CAT mutants with $\mu = 200$. Values shown here are IC90 of the mutant divided by IC90 of the WT for each parameter combination. Linear correlations are computed between $\log_{10}$-transformed parameter values and $\log_{10}$-transformed fold-increase in IC90 for each cell type.
